# Health Care Worker Preferences and Perspectives on Doses per Container for 2 Lyophilized Vaccines in Senegal, Vietnam, and Zambia

**DOI:** 10.9745/GHSP-D-20-00112

**Published:** 2020-12-23

**Authors:** Natasha Kanagat, Kirstin Krudwig, Karen A. Wilkins, Sydney Kaweme, Guissimon Phiri, Frances D. Mwansa, Mercy Mvundura, Joanie Robertson, Debra Kristensen, Abdoulaye Gueye, Sang D. Dao, Pham Q. Thai, Huyen T. Nguyen, Thang C. Tran

**Affiliations:** a JSI Research & Training Institute, Inc., Arlington, VA, USA.; b Consultant, JSI Research & Training Institute, Inc., Arlington, VA, USA.; c Republic of Zambia Ministry of Health, Lusaka, Zambia.; d PATH, Seattle, WA, USA.; e PATH, Geneva, Switzerland.; f PATH, Dakar, Senegal.; g PATH, Hanoi, Vietnam.; h National Institute of Hygiene and Epidemiology, Hanoi, Vietnam.

## Abstract

**Introduction:**

Limited information exists on health care workers’ (HCWs) perceptions about use of multidose vaccine vials and their preferences about doses per container (DPC). We present findings from qualitative studies conducted in Senegal, Vietnam, and Zambia to explore HCWs’ behavior regarding opening vials and their perceptions and preferences for the number of doses in vials of BCG and measles-containing vaccine (MCV). Zambia and Senegal currently offer MCV in 10-dose vials and BCG in 20-dose vials; 10-dose vials are used for both vaccines in Vietnam. Unused doses in vials of these reconstituted vaccines must be discarded within 6 hours.

**Methods:**

Key informant interviews (KIIs) were conducted with frontline HCWs in Senegal, Vietnam, and Zambia. In Senegal and Vietnam, the KIIs were conducted as part of broader formative research; in Zambia, KIIs were conducted in control districts using 10-dose MCV vials only and in intervention districts that switched from 10- to 5-dose vials during the study. During analysis, themes common to all 3 countries were synthesized. Critical themes relevant to country contexts were also examined.

**Results:**

HCWs in all 3 countries preferred containers with fewer doses for BCG and MCV to reduce wastage and increase the likelihood of vaccinating every eligible child. HCWs in Senegal and HCWs using 10-dose vials in Zambia reported sending unvaccinated children away because not enough children were present to warrant opening a new vial. In Vietnam, where sessions are typically held monthly, and in Zambia when the 5-dose vials were used, almost all HCWs reported opening a vial of MCV for even 1 child.

**Discussion:**

HCWs prefer vials with fewer DPC. Their concerns about balancing coverage and wastage influence their decisions to vaccinate every eligible child; and their perspectives are crucial to ensuring that all target populations are reached with vaccines in a timely manner.

## Abstract (precis)

When providing immunization services, health care workers balance the mandate of achieving high coverage with limiting vaccine wastage. Workers in 3 countries said that containers with fewer vaccine doses for measles and BCG would enable them to immunize all children who present, while reducing concerns about wasting vaccine.

## Teaser

**Key Findings:**

- Health care workers (HCWs) in all 3 countries preferred containers with fewer doses for reconstituted vaccines such as BCG and measles-containing vaccine.
- HCWs believed that containers with fewer doses of these vaccines could reduce wastage and missed vaccination opportunities.
- HCWs were more willing to open a vial for every eligible child when using containers with fewer doses.

**Key Implications:**

- Policy makers should consider HCW perspectives when deliberating a change in policy on vial size since HCWs have to balance concerns about open vial wastage with the guidance to open a vial to vaccinate every eligible child.
- Program managers should consider shifting to containers with fewer doses for vaccines without preservatives to assuage HCW concerns about opening vials for every eligible child while managing wastage.

Résumé en français à la fin de l'article.

## INTRODUCTION

Many vaccines administered in low- and middle-income countries are purchased in multidose vials (MDVs) and can contain between 2 and 20 doses per vial or container.^1^ ^,^ ^2^ Several countries buy vaccines in MDVs because compared with single-use vials, MDVs sell at a lower price per dose; require lower cold chain, storage, and transport capacity; and generate less waste.^3^ Some vaccines contain preservatives, whereas others do not. Under the World Health Organization’s (WHO’s) multidose vial policy,^4^ remaining doses in open vials of vaccines with preservatives can be used for up to 28 days after opening, as long as storage and proper handling conditions are met. However, vaccines without preservatives must be used in a much shorter time frame. Vaccines such as BCG, measles-containing vaccine (MCV), and yellow fever vaccines do not contain preservatives, and they must be discarded within 6 hours of reconstitution or at the end of a session, whichever comes first. Health care workers (HCWs) in low- and middle-income countries who administer these vaccines to their target populations are therefore responsible for deciding when to open a vial, knowing that if not all doses are used within a short frame of time, they will have to be discarded, resulting in open-vial wastage.

This article focuses on vaccines without preservatives. HCWs have to balance the expectation that they will vaccinate every child with the concerns about open-vial wastage. Open-vial wastage tends to increase with vaccines that have more doses per container (DPC) when the immunization session sizes are small.^5^ Limited information exists on HCWs’ opinions about the desired DPC and how DPC informs their decisions on when to vaccinate. Studies by Wallace et al.^6^ and Hutchins et al.^7^ suggest that HCWs’ hesitancy to open a multidose vaccine vial to avoid vaccine wastage contributed to missed opportunities for vaccination (MOVs). HCWs’ behavior regarding opening vials is critical to addressing MOVs, which emphasizes efforts to reach eligible children at all immunization sessions, including outreach, to identify and reduce opportunities missed at the health facility level on a day-to-day basis. MOVs can result in inadequate protection against disease.

The qualitative studies described here (see Methods section) were part of a larger multicountry project to improve the evidence base on HCWs’ decision making relative to DPC. This article focuses on qualitative findings on HCWs’ perspectives on BCG and MCV, obtained through formative research in Senegal and Vietnam and a prospective study in Zambia. The project also conducted household surveys to examine immunization coverage, administered facility surveys to conduct cost-effectiveness analyses, and studied routine administrative data from facilities to assess vaccine wastage. The findings from those studies are forthcoming in other journals.

In this article, we report on the BCG and MCV vaccines since they are common to all 3 countries, are supplied in MDVs, and must be discarded after 6 hours. Zambia and Senegal use a 10-dose measles-rubella (MR) vial and a 20-dose BCG vial. Vietnam uses 10-dose MCV (both measles and MR) and BCG vials; measles first dose is given at 9 months and MR vaccine (second dose) is given at 18 months.

In Senegal, the MR first dose is given at 9 months and is coadministered with yellow fever vaccine. The MR second dose is given at 15 months. All routine childhood immunizations are given during fixed and outreach sessions. Health facilities conduct fixed sessions that are held anywhere from daily to monthly, depending on the catchment population and size of the facility. Outreach sessions vary in frequency, depending on the number of outreach locations, availability of staff to conduct outreach, and other factors. In Vietnam, fixed sessions are held once or twice a month in most of the country, although in some districts immunization is organized once weekly. Outreach sessions are not conducted everywhere, and they vary in frequency where offered. In Zambia, MR is given at 9 months and 18 months of age. Routine childhood immunizations in Zambia are given during fixed and outreach sessions. Health facilities hold sessions anywhere from daily to monthly depending on the catchment population, size of the facility, availability of staff to conduct outreach, and other factors.

In Zambia, the guidance is to open a vial for every eligible child, and WHO’s multidose vial policy is followed: Vaccines with preservatives can be kept for up to 28 days, while vaccines without preservatives (i.e., BCG, MCV, and yellow fever) must be discarded 6 hours after reconstitution or at the end of an immunization session, whichever comes sooner.

Vaccines with preservatives can be kept for up to 28 days, while vaccines without preservatives must be discarded 6 hours after reconstitution.

In Senegal, the national level does not give guidance to health facility staff on how many eligible children must be present at a session before HCWs can open a vial. As in Zambia, WHO’s multidose vial policy is followed.

In Vietnam, sessions are held monthly in most health facilities; therefore, the policy for all vaccines is to discard all remaining doses in opened vials at the end of each session.

## METHODS

The qualitative study in Senegal was conducted as part of a broader formative research study (Box). Sixty health facilities (HFs) were included in the study and 1 HCW per health facility participated in qualitative interviews. Health facilities were selected through stratified random sampling based on rural or urban locations, size of birth cohort served, and distance from the district vaccine store. The study was conducted in 2 regions, Louga and Ziguinchor, selected because their vaccine coverage rates are below national coverage rates.

BOX Introduction to the Dose Per Container Partnership Project Under Which the Studies Presented in the Article Were Conducted

The global effort to protect all people from vaccine-preventable diseases has historically leveraged multidose containers in low- and middle-income countries to offer lower prices and reduce the constraints on cold chain space. However, as newer, more expensive vaccines are introduced in multidose formats, the burden of cost efficiency potentially moves from the national-level to the health care worker.

To achieve maximum utilization of every dose in a vial and depending on the country’s policies, health care workers need to be strategic about when to open a vial and be diligent about how they care for open vials. In addition, they have to be more active with community outreach and communication to ensure optimal attendance and timely immunization. For these reasons, the number of doses per container (DPC) can hinder a country’s ability to achieve timely and equitable coverage including reaching the urban poor or rural remote. DPC can also influence additional factors like vaccine safety, system costs, supply chain, and wastage.

The Dose Per Container Partnership (DPCP) was a multicountry project that aimed to support vaccine product and program decision making to include considerations of DPC to optimize equitable, timely, safe, and cost-effective coverage.

The Partnership implemented country-level research in several countries, including Senegal, Zambia and Vietnam, to generate new evidence on the impact of DPC decisions on an immunization system, to explore current decision making on DPC options, and to inform country and global decisions on vaccine procurement. The Partnership has produced case studies on decision making and multicountry research, as well as videos, resource guides, and briefs on various aspects of DPC.

DPCP is funded by the Bill & Melinda Gates Foundation, led by JSI Research & Training Institute, Inc. and jointly implemented in partnership with PATH, the Clinton Health Access Initiative, the Highly Extensible Resource for Modeling Event-Driven Supply Chains modeling team, and the International Vaccine Access Center.

The qualitative study in Vietnam was conducted as part of a broader formative research study that included 30 health facilities. Thirty HCWs (1 per HF) participated in the qualitative interviews. The study was conducted in 4 provinces in 2 regions in Vietnam: Dien Bien, Tuyen Quang, and Yen Bai in Northern Region, and Dak Lak in the Central Highlands Region. Dak Lak Province was chosen specifically because the immunization session is organized once every week, whereas in the other sites the immunization sessions are conducted once or twice a month. The researchers wanted to assess whether HCWs’ perspectives differed across sites with different session frequencies. In Northern Region, 2 districts in each province and 3 communes in each district were selected. In Central Highlands Region, the researchers focused on the one district that was conducting weekly immunization, and they included 12 communes from that district and therefore oversampled in the district with weekly immunization. Thirty HFs were purposively sampled, taking into account coverage and different service delivery models. In the Northern Region, health facilities were selected either because they had coverage rates below 90% or the lowest coverage in the district. In some cases, even if they had the lowest coverage rates in the district, their coverage was over 90%. Most HFs in the Northern Region are in rural settings, whereas the facilities in the Central Highlands Region contained more of a mix of rural, peri-urban, and urban sites. In Vietnam, HCWs were asked about measles and MR separately, because their vaccine schedule requires measles for first dose and MR for second dose.

The qualitative study in Zambia was conducted as part of a broader implementation research study. In Zambia, 90 HCW interviews were conducted across 14 districts in 2 provinces. For the implementation research, all districts were paired according to average population size per HF and the number of HFs. From each pair, 1 district was randomly assigned to the intervention, while the other district served as the control. During the implementation period, all HFs in the intervention district received 5-dose vials of MCV, while the control group continued to use the 10-dose vials. The HFs receiving the 5-dose vials were oriented to the new vial size, but no other technical support was offered over the 1-year implementation period, to minimize influencing the HCWs’ behavior. The project wanted to keep the settings as neutral as possible to allow us to observe differences in behavior at endline. HCW interviews for the qualitative study were conducted at baseline (32), midline (16), and endline (42).

Interview teams verified that respondents from all 3 countries were responsible for providing routine immunizations to ensure they had an understanding of immunization service delivery. HFs were selected to ensure representation across large, medium-sized, and small facilities in urban and rural locations within each district.

At baseline, none of the districts in Zambia had switched over to the 5-dose vials, so all 32 interviews were conducted with HCWs who were using the 10-dose vials. The midline and endline interviews, however, were conducted after the districts were divided into those receiving 10-dose vials and those receiving 5-dose vials, so midline and endline interviews were held with HCWs in districts receiving 5-dose vials to document their experiences using the new presentation.

In all 3 countries, contracted local data collectors gathered data with oversight and supervision by the organization leading the country study. Data analysis for each country was done separately. For Senegal and Vietnam, responses to the qualitative surveys were analyzed in Excel. For the questions with predefined response options, the responses were counted based on the response options. For the open-ended questions, the key themes from the responses were also tabulated and reported. For Zambia, all transcripts were uploaded into NVivo 11, a qualitative data management software. The qualitative team generated an initial set of codes derived from the research questions to analyze the data. All codes were accompanied by code definitions. The initial set of codes comprised major thematic categories, which were refined through analysis, and subcategories (i.e., subcodes) were developed through iterative analysis. For this article, all the country reports and briefs generated from the separate analysis were then reviewed to identify major themes common to all 3 countries and summarize findings. We also highlight country-specific findings.

The formative research studies in Senegal and Vietnam were determined by PATH’s Research Determination Committee not to be human subjects research. Approvals were obtained from the Ministry of Health/Senegal Ethics Committee and the Ethics Committee of the Vietnamese National Institute of Hygiene and Epidemiology. The implementation study in Zambia received ethical approval from the Biomedical Research Ethics Committee of the University of Zambia.

## RESULTS

### HCW Perceptions on Reducing Missed Opportunities When Using 5-Dose Vials

All HCWs were asked (1) whether they vaccinated every eligible child each time the child was at the health facility; (2) if MCV and BCG were offered at every session; and (3) if they opened a vial of these vaccines at a session irrespective of the number of eligible children present. These questions were asked to assess whether concerns about opening a vial for only 1 child or a few children resulted in HCWs either not opening the vial or waiting for a minimum number of children being present to justify opening it.

All HCWs were asked whether they vaccinated every eligible child and if they opened a vial of vaccines without preservative irrespective of how many children were present.

In Senegal, MCV, BCG, and yellow fever vaccines were not offered at every immunization session. Most respondents said that at least 5 children had to be present for MCV and yellow fever vaccine and 10 children had to be present for BCG before they would open a vial. When fewer children attended a session, HCWs asked them to return on another date when the next session was scheduled. In Senegal, the majority of HCWs recalled turning away a caregiver and child from a vaccination session at least once in the last 3 months. Similar findings were also observed for yellow fever vaccine and MR, which is also in 10-dose vials and is coadministered with MR.

*I programmed them for the next session, it’s for tomorrow. I have recorded their coordinates [location] and the relays [community health workers] take care to find them, and if they do not come, we call them on the telephone.—Senegal*

In Vietnam, the majority of facilities conduct sessions once a month, and all vaccines are offered at each session. Therefore, the majority of HCWs reported that they opened a vial for every child and were willing to open a vial during sessions when only 1 child was eligible, regardless of potential wastage. Most HCWs in Vietnam did not recall sending children away during an immunization session because not enough children were present to warrant opening a vaccine vial. The few HCWs who recalled sending children away mentioned that they advised the caregivers to bring the child back for the next session.

*Sending children away without vaccination takes time because the caregivers have to bring their children back for the other immunization session.—Vietnam*

In Zambia, over half of HCWs using 10-dose vials of MCV reported waiting for a minimum of 5 children before opening a vial, and a minimum of 10 children to open a vial of BCG. However, when HCWs using 5-dose vials were asked about their practices, they replied that they were less concerned about MCV wastage and felt more comfortable opening vials to vaccinate children. Most HCWs in Zambia using the 5-dose vial stated that they opened a vial each time an eligible child presented during a session and did not wait for a minimum number of children.

*We can open the vial even when we have two children, we only lose three doses as compared to the time we were using ten-dose vials, this would make us lose eight doses.—Zambia 5-dose vial district*

In districts that continued to use the 10-dose MCV vial in Zambia, the majority of HCWs recalled turning away a child from a vaccination session at least once in the past 3 months. In the Zambian districts using 5-dose vials of MCV, very few HCWs reported turning children away. Neither group of respondents had a system to track whether the children turned away were brought back to the facilities for vaccinations in the future.

*Yes, because everyone is concerned on reducing the vaccine wastage. It is a reason why mothers are sent back and asked to come a different day when there are enough children to open the vial. This is so because everyone wants to reduce the wastage.—Zambia 10-dose vial district*

### Balancing Coverage and Wastage

All HCWs were asked whether their supervisors considered coverage rates or wastage rates more important, since the supervisors’ belief would influence what the HCWs placed more emphasis on. The belief also could affect HCWs’ behavior if they offered certain vaccines at specific times to ensure adequate numbers of children to minimize wastage. Most HCWs from Senegal, Vietnam, and Zambia stated that their supervisors deemed coverage more important than wastage. However, if wastage rates were deemed higher than expected, HCWs reported that supervisors offered suggestions and strategies to mitigate wastage.

All HCWs were asked whether their supervisors considered coverage rates or wastage rates more important.

In Senegal, most HCWs knew the target wastage rates for each vaccine. Knowing the target affected which vaccines were offered at each session. To minimize wastage rates, HCWs reported not offering vaccines like BCG and MCV at each session. When asked how they ensured that a certain number of children were present for the BCG and MCV sessions, they replied:

*Collect 10 children for BCG, MR [measles rubella], before opening the bottle [vial], using relays and badjene ngokh [community health workers] who will bring the children and remind parents of the RV [vaccination session].—Senegal*

Most HCWs in Senegal and the 10-dose districts of Zambia also reported that MCV and BCG vaccines were not offered at every session. The reason for not offering these vaccines was to increase session sizes for these specific vaccines to reduce wastage.

In Vietnam, the session frequency determined whether all vaccines were offered at every session. At sites where immunization was offered once or twice a month, all vaccines were offered every time. At sites where immunization was offered more frequently, not all vaccines were offered every time, to avoid vaccine wastage.

*BCG, measles, and Japanese encephalitis (JE) vaccines are injected once every 2 weeks. DPT and MR vaccines are injected once per month. It is because the number of children who need these vaccines is less than that of other vaccines.—Vietnam*

By contrast, in the Zambian districts using 5-dose vials of MCV, the majority of HCWs reported offering MCV at every fixed session regardless of the number of children. The HCWs in the 5-dose districts did not know their wastage rates, but they believed that wastage had diminished with their use of the 5-dose vials, and they were therefore less concerned about opening the MCV 5-dose vial for fewer children. In all 3 countries, BCG was given on specific days, such as at postnatal sessions at health facilities or hospitals, or on a designated day per month, to ensure that a large number of children would be present and wastage could be limited.

*Yes there are days when MCV and BCG is not given during outreach, for example you find two babies who have been delivered. Are you going to open that vial for BCG just for those two? So in such cases we explain to them that we can’t offer them BCG or MCV at that particular moment. We then advise them to come to the center, especially on the last Thursday of the month.—Zambia 10-dose district*

### What Are HCWs’ Preferences for DPC?

All HCWs expressed a preference for a different vial size of BCG and MCV with fewer DPC. In Senegal, most HCWs preferred fewer DPC for these vaccines to reduce wastage, and many said that this could help to address the challenge of needing enough eligible children to warrant opening a vial, prevent dropouts, and provide services to hard-to-reach children. A couple of HCWs felt that fewer DPC might pose challenges for storage or transportation of vials. In Zambia, HCWs from the 5-dose districts also expressed a preference for fewer DPC vials for MCV; none of them wanted to return to using 10-dose vials. The majority of HCWs in the 5-dose districts preferred 5-dose vials, and the rest preferred fewer than 5 doses. In Vietnam, for BCG and MCV (currently in 10-dose vials), the majority of HCWs expressed a preference for a 1-dose vial, followed by a 5-dose and a 2-dose vial (Table).

*Because of the mobile population here we often lose sight of children—having single dose presentations would permit us to vaccinate each child who presents.—Senegal*

*If vaccine was packed in single dose per vial, we could conduct vaccination in more days per month instead of doing in 1 day.—Vietnam health facilities conducting weekly sessions*

*It has made things easier for us in that you do not have to worry about babies not being immunized; it’s rare that we miss out any child. It has made our work easier; our minds are free that we are doing our job [immunizing] unlike the BCG.—Zambia 5-dose vial district*

**TABLE.** Summary Findings on Vaccine Doses per Container and Health Care Workers’ Perceptions and Practices In 3 Countries

**Theme**	**Summary Findings**
HCW perceptions on reducing missed opportunities when using 5-dose vials	Senegal: MCV, BCG, and yellow fever vaccines were not offered every time immunization sessions were held. During immunization sessions, HCWs reported that they waited for a minimum number of children before opening these vaccines. HCWs recalled turning away a caregiver and child at least once in the past 3 months. Vietnam: Due to Vietnam’s session schedules, which are mostly once a month, most HCWs did not wait for a minimum number of children before opening vials. They did not recall turning away children in the past 3 months. Zambia: In the districts using the 10-dose vials, HCWs waited for a minimum of 5 children to open an MCV vial and 10 children to open a 20-dose BCG vial. In the districts using the 5-dose vials, HCWs opened a vial each time an eligible child presented and did not report turning away a child.
Balancing coverage and wastage	In all 3 countries, although coverage was considered more important, HCWs reported that wastage was tracked very closely and they knew they had to minimize wastage as much as possible. In Zambia and Senegal, HCWs did not offer MCV, BCG, or yellow fever vaccines (Senegal only) at every session due to concerns about wastage. The intent was to increase session sizes for these specific vaccines as a way of reducing open vial wastage. In the facilities offering 5-dose vials in Zambia, HCWs believed that their wastage was lower, and they expressed less concern about opening the vial for fewer children compared with the facilities using the 10-dose vials.
HCWs’ preferences for DPC	All HCWs expressed a preference for fewer DPC for BCG and MCV (and yellow fever for Senegal) to allow them to vaccinate eligible children, prevent dropouts, and not worry about wastage. No HCWs in the districts in Zambia that used the 5-dose vials during implementation wanted to return to using the 10-dose vials.

Abbreviations: BCG, bacille Calmette-Guérin; DPC, doses per container; MCV, measles-containing vaccine; HCW, health care worker.

All HCWs expressed a preference for a different vial size of BCG and MCV with fewer DPC.

## DISCUSSION

This multicountry study demonstrates that waiting for a minimum number of children before opening a vial of BCG and MCV could result in MOVs. Eliminating or greatly reducing MOVs is critical to achieving the Global Vaccine Action Plan 2011-2020 goal of “90% national coverage and 80% in every district or equivalent administrative unit, for all vaccines in the national immunization schedule.”^8^ HCWs serve as critical intermediaries between communities and the health system, and they regularly make decisions about the management and delivery of vaccine services to achieve recommended coverage levels. Although many factors contribute to MOVs, it is vital to recognize the role of vials with fewer DPC for reconstituted vaccines in reducing instances of MOVs. As is seen in Vietnam, with the variation of timing and vaccines offered during immunization sessions, high-level structural decisions are made to balance coverage and wastage. This study also highlights the importance of providing HCWs with options that do not require sacrificing vaccination coverage *or* having high wastage. Current practices create a tension between expectations and ground realities, obligating HCWs to offer lifesaving vaccines infrequently, turn away children, or risk not meeting expectations on wastage. This finding supports the conclusions of Wallace et al.^9^ that HCWs either take active measures to reduce wastage or feel some conflict when wastage is high. Separate quantitative analyses from this project confirm HCWs’ perceptions that fewer DPC will likely increase coverage children and reduce wastage.^10^ In Zambia, facilities using 5-dose vials had 47% lower wastage rates compared with those using 10-dose vials. An increase in coverage of MR first and second dose respectively by 5% and 3.5% in the districts using 5-dose vials was attributable to the intervention (i.e., the use of the 5-dose vials).

It is vital to recognize the role of vials with fewer DPC for reconstituted vaccines in reducing MOVs.

HCWs from all countries also reported turning away children if not enough children were present to warrant opening a vial, and in many cases, no system was in place to ensure that these children would be vaccinated later. This practice goes against WHO recommendations that vaccination programs include daily opportunities for vaccination with all vaccines, offering vaccination at every contact, including screening at curative consultations, even if there is only 1 child.^11^ This behavior also represents a MOV, requiring additional effort by caregivers and HCWs to follow up and increasing the chance that the child will not receive BCG or MCV.^12^ Similar findings have been documented in other low- and middle-income countries where BCG and MCV are offered less frequently than vaccines that do not have to be discarded after 6 hours of reconstitution, as a way to increase the number of children present per session before opening a vial.^6^ ^,^ ^13^

Our findings are pertinent to current discussions on session sizes during the coronavirus disease (COVID-19) pandemic. Due to the pandemic, WHO guidance recommends frequent routine immunization sessions of smaller size to reduce the risk of spreading the virus.^14^

As more countries consider changing their DPC for different vaccines, decisions should take HCWs’ perspectives into account. This approach is not always the norm. Other DPCP case studies on decision making on DPC in Ghana, Benin, Côte d’Ivoire, and the Democratic Republic of Congo showed that HCWs’ perspectives were notably absent.^15^ ^–^ ^17^

As more countries consider changing their DPC for different vaccines, decisions should take HCWs’ perspectives into account.

We recommend that future research continue to explore the causal links between HCWs’ practices related to vaccine wastage and their impact on vaccination coverage, MOVs, and cost implications. We also recommend additional research on HCWs’ preferences in other countries and settings to expand the body of evidence regarding HCWs’ decision making about opening vials.

### Limitations

This study had limitations, including differences in study design between countries and different criteria used to select health facilities and key informants. Data analysis was also done by separate teams. In 1 country, respondents were purposefully selected, which may limit the generalizability of the results. However, the large sample used for the qualitative interviews in all 3 countries ensured that we got an appropriate and adequate number of respondents whose views likely represent those of the larger population of HCWs. In Zambia, we collected data at 3 different times to enable us to document behavior change in HCWs, especially in the districts that switched to using the 5-dose vials. The researchers tried to address these differences by ensuring that data on priority themes were collected across all countries, and that research protocols, data collection tools, and draft reports were shared among teams to establish a level of consistency in the data being collected and analyzed. Another limitation is that this study focused on relatively low-performing districts. However, our findings are likely also applicable to high-performing districts given that public-sector resources are always limited and that striking a balance between vaccinating every child and limiting wastage will be a difficult decision for HCWs in both high- and low-performing districts.

## CONCLUSION

This 3-country study contributed evidence on HCWs’ perceptions and preferences with regard to various DPC options for reconstituted vaccines. The results suggest that when balancing the mandate to achieve high coverage and reduce vaccine wastage, HCWs have to decide when to open a vial with more DPC. In all 3 countries, high coverage rates were considered more important than not exceeding wastage targets. However, the desire to control or reduce wastage rates, although secondary, was considered important and did influence HCW behavior. As shown, in the Zambia 5-dose districts, HCWs reported offering MCV at every fixed session—a change from when they were using the 10-dose vials. In Senegal, vaccines eligible for use for 28 days after opening were offered at every session, unlike vaccines that had to be discarded within 6 hours of reconstitution. HCWs in districts that received the 5-dose vials of MCV reported that they were more likely to open a vial for 1 child than they had been when they had 10-dose vials, representing a possible solution to minimizing MOVs. This change in behavior was influenced by their reduced fear of wastage when opening a vial with fewer DPC.

## En français

**Préférences des professionnels de la santé et perspectives sur les doses par flacon de deux vaccins lyophilisés au Sénégal, au Vietnam et en Zambie**

**Messages clés**

Lorsqu'ils fournissent des services de vaccination, les agents de santé trouvent un équilibre entre la nécessité d'atteindre une couverture élevée et celle de limiter le gaspillage de vaccins. Les travailleurs de 3 pays ont déclaré que des récipients contenant moins de doses de vaccin contre la rougeole et le BCG leur permettraient de vacciner tous les enfants qui se présentent, tout en réduisant les inquiétudes quant au gaspillage de vaccin.

**Résumé** **Introduction:** Il existe peu d'informations sur les perceptions des agents de santé (AS) concernant l'utilisation des flacons de vaccins multidoses et leurs préférences en matière de doses par flacon (DPF). Nous présentons les résultats d'études qualitatives menées au Sénégal, au Vietnam et en Zambie pour étudier le comportement des agents de santé concernant l'ouverture des flacons et leurs perceptions et préférences quant au nombre de doses dans les flacons de BCG et de vaccin contre la rougeole. La Zambie et le Sénégal utilisent actuellement le vaccin contre la rougeole en flacons de 10 doses et le BCG en flacons de 20 doses; des flacons de 10 doses sont utilisés pour les deux vaccins au Vietnam. Les doses inutilisées des flacons de ces vaccins reconstitués doivent être jetées dans les 6 heures.

**Méthodes:** Des entretiens avec des informateurs clés ont été menés avec des agents de santé de première ligne au Sénégal, au Vietnam et en Zambie. Au Sénégal et au Vietnam, les entretiens ont été menées dans le cadre d'une recherche formative plus large; en Zambie, les entretiens ont été menées dans les districts de contrôle en utilisant uniquement des flacons de 10 doses de vaccin contre la rougeole et dans les districts d'intervention qui sont passés de flacons de 10 à 5 doses au cours de l'étude. Au cours de l'analyse, les sujets communs aux trois pays ont été synthétisés. Les sujets critiques pertinents pour les contextes nationaux ont également été examinés.

**Résultats:** Les travailleurs de la santé des trois pays ont préféré des récipients contenant moins de doses de BCG et de vaccin contre la rougeole afin de réduire le gaspillage et d'augmenter la probabilité de vacciner chaque enfant éligible. Les agents de santé au Sénégal et les agents de santé utilisant des flacons de 10 doses en Zambie ont déclaré avoir renvoyé des enfants non vaccinés parce qu'il n'y avait pas assez d'enfants présents pour justifier l'ouverture d'un nouveau flacon. Au Vietnam, où les séances ont généralement lieu tous les mois, et en Zambie, où les flacons de 5 doses ont été utilisés, presque tous les travailleurs de la santé ont déclaré avoir ouvert un flacon de Rougeole, même pour un seul enfant.

**Discussion:** Les agents de santé préfèrent les flacons contenant moins de dose par flacon. Leur souci d'équilibrer la couverture et le gaspillage influence leurs décisions de vacciner chaque enfant éligible; et leur point de vue est crucial pour garantir que toutes les populations cibles soient vaccinées en temps voulu.

## Notes

**Peer Reviewed**

**First published online:** December 4, 2020
